# Exacerbation of Behcet’s Disease and Pyoderma Gangrenosum Following COVID-19 Infection: A Case Report

**DOI:** 10.7759/cureus.49386

**Published:** 2023-11-25

**Authors:** Luis F Álvarez Pérez, Salvador Vila

**Affiliations:** 1 Department of Medicine, University of Puerto Rico, Medical Sciences Campus, San Juan, PRI

**Keywords:** autoimmune disease, cytokine storm, behcet's disease, covid-19, pyoderma gangrenosum

## Abstract

Behcet’s disease (BD) and pyoderma gangrenosum (PG) are rare autoimmune inflammatory diseases that have been reported to relapse following COVID-19 infection. BD is a multisystemic syndrome that may involve multiple body organs. PG is a skin disease that can be a part of the skin involvement of BD. We report a 33-year-old woman with BD and PG who developed headaches, arthralgias, and rapidly progressive painful skin ulcers after COVID-19. She had not complained about BD or PG symptoms for two years prior to admission. Treatment at admission comprised infliximab 560 mg every eight weeks, azathioprine 50 mg daily, and low-dose aspirin. Due to the suspicion of neuro BD and the rapid appearance and progression of the ulcers, she was treated with intravenous (IV) methylprednisolone 1000 mg daily three times followed by prednisone at 1 mg/kg/day. Azathioprine was increased to 100 mg bid. Local ulcer care was provided. She was discharged home on the eighth hospital day. The arthralgias were completely gone, and the headaches and skin ulcers had improved. Six months after discharge, she was off prednisone and continued infliximab and azathioprine. She had no headaches or joint pains, and the ulcers had completely healed. One year after admission, BD and PG signs and symptoms had completely disappeared. This case highlights the importance of recognizing that autoimmune diseases may exacerbate COVID-19. Timely management is crucial to prevent complications and morbidity. To our knowledge, this is a rare case report describing BD and PG exacerbation following COVID-19.

## Introduction

Behcet’s disease (BD) is a rare inflammatory disease most frequently seen in the population of the Middle and Far East such as Turkey, Iran, Israel, and Japan. In these countries, males are affected more frequently than females. In the US, the frequency of BD was calculated at 5/100,000, the mean age at diagnosis was 48.7±16.3 years, female to male ratio was 3.8:1, and 66.7% were White [1]. BD attacks multiple body organs including the skin. Pyoderma gangrenosum (PG) is a very rare manifestation of skin involvement in BD.

PG occurs as an independent skin disease or in association with other autoimmune diseases, infections, hematologic disorders, and traumatic events. Prevalence in the US is calculated at around 6/100,000 adults. It is around 1.8 times more common in females [2]. The diagnosis is based on the clinical characteristics of the ulcers and pathological findings. When associated with an autoimmune disease it is most commonly associated with inflammatory bowel disease, but it has also been reported to occur in BD [3-15].

COVID-19 infection has been associated with exacerbation of autoimmune disorders [16-20]. Relapse of previously controlled BD and PG has been reported following COVID-19. We report a young woman with BD and PG who had been in remission for two years, and who, after infection with the virus, had a severe relapse of PG and BD that required hospital admission and aggressive treatment.

## Case presentation

A 33-year-old woman presented to the Emergency Department (ED) due to the development of a rapidly progressive and painful ulcer in the right medial tibia. The skin lesion started as a raised reddish-brown colored blister that over the course of a few days ulcerated and expanded. The border of the ulcer was raised, erythematous, and it was oozing a blood-tinged, serous fluid. She also complained of general malaise, severe headache, fever, chills, arthralgias, and xerostomia. She did not report oral or genital ulcers, photophobia, abdominal pain, diarrhea, or confusion.

One month prior to the ulcer’s development, she experienced debilitating headaches and musculoskeletal pain that hampered her daily activities. She did a COVID-19 PCR test that confirmed her suspicion of infection. She had debilitating headaches and arthralgias but did not require antiviral medication or hospitalization. Her symptoms improved four days after starting. However, seven days after the COVID-19 diagnosis she observed the emergence of a raised reddish lesion, which, within days, progressed into a painful ulcer. Severe headaches and arthralgias returned at the same time the skin lesion appeared. 

The woman was diagnosed with BD in 2017. This diagnosis was based on the presence of recurrent aphthous oral ulcerations, monthly occurring vaginal ulcers for a period of over 12 months, a positive pathergy test, and recurrent episcleritis. At the time, she had painful lower extremity ulcers, which, upon biopsy, were found to be neutrophilic dermatosis consistent with a diagnosis of PG. She started high-dose corticosteroids, infliximab, and azathioprine achieving remission of symptoms over time. Corticosteroids were slowly tapered and discontinued, and she remained on infliximab 560 mg every eight weeks and azathioprine 50 mg daily, achieving complete resolution of her disease symptoms two years before her hospital admission.

In the ED, her blood pressure was 121/66 mmHg, heart rate was 67 beats per minute, respiratory rate was 18, and temperature was 36.70°C. She had a 4 cm diameter ulcer in the right lateral buttock and an 8 cm diameter ulcer in the right lower leg medial tibial side. The ulcers had irregular, raised, undermined erythematous borders. She had tenderness in the second and third proximal interphalangeal joints, without swelling, of both hands. Lymphadenopathies were not palpable in the neck, axillary, or inguinal area. She did not have aphthous ulcers, eye erythema, or photosensitivity. The cardiac, pulmonary, neurologic, and abdominal exams were unremarkable.

The initial laboratory findings are presented below in table form (Table 1 and Table 2).

**Table 1:** Summary of laboratory workup at initial presentation CBC, complete blood count; WBC, white blood cell; PLT, platelet; Ab, antibody; ANA, antinuclear antibodies; anti-dsDNA, anti-double-stranded DNA; RNP, ribonucleoprotein; β2GPI, beta-2 glycoprotein I; PR3, anti-Pr-3 antibodies; MPO, myeloperoxidase antibodies

Laboratory	Result	Reference range with units
CBC		
WBC	8.6	4-10.04 X10^-3^/uL
Hemoglobin	10.1	11.2-15.7 g/dL
PLT	405	163-369 X10^-3^/uL
Acute phase reactants		
Erythrocyte sedimentation rate	22	0-20 mm/hr
C-reactive protein quantitative	1.52	<5 mg/dL
Serology		
ANA	Positive	Negative
Anti-dsDNA Ab	Negative	<10 U/mL
Anti-Smith Ab	0.9	<7 U/mL
Anti-RNP Ab	<0.5	<5 U/mL
Anticardiolipin Ab	<2.0	<20.0 U/mL
β2GPI	<1.0	<20.0 U/mL
Lupus anticoagulant	Negative	Negative
PR3 Ab	<1.0	<1.0 AI
MPO Ab	<1.0	<1.0 AI
Complement 3	91	90-180
Complement 4	22.6	10-40
Anti-RO Ab	1	<7
Anti-La Ab	0.7	<7

**Table 2:** Summary of laboratory workup at initial presentation CMP, comprehensive metabolic panel; BUN, blood urea nitrogen; AST, aspartate aminotransferase; ALT, alanine aminotransferase

Laboratory	Result	Reference range with units
CMP		
Glucose	86	70-99 mg/dL
BUN	9.1	10-26 mg/dL
Creatinine	0.56	0.7-1.5 mg/dL
Sodium	138	135-145 mEq/L
Potassium	4.2	3.5-5 mEq/L
CO_2_	27	24-32 mEq/L
ALT	12	0-45 U/L
AST	16	0-45 U/L
Alkaline phosphatase	76	30-115 U/L
Total bilirubin	0.27	0.2-1.3 mg/dL
Calcium	8.8	8.5-10.5 mg/dL
Albumin	4.1	2.6-5.2 g/dL

**Figure 1:**
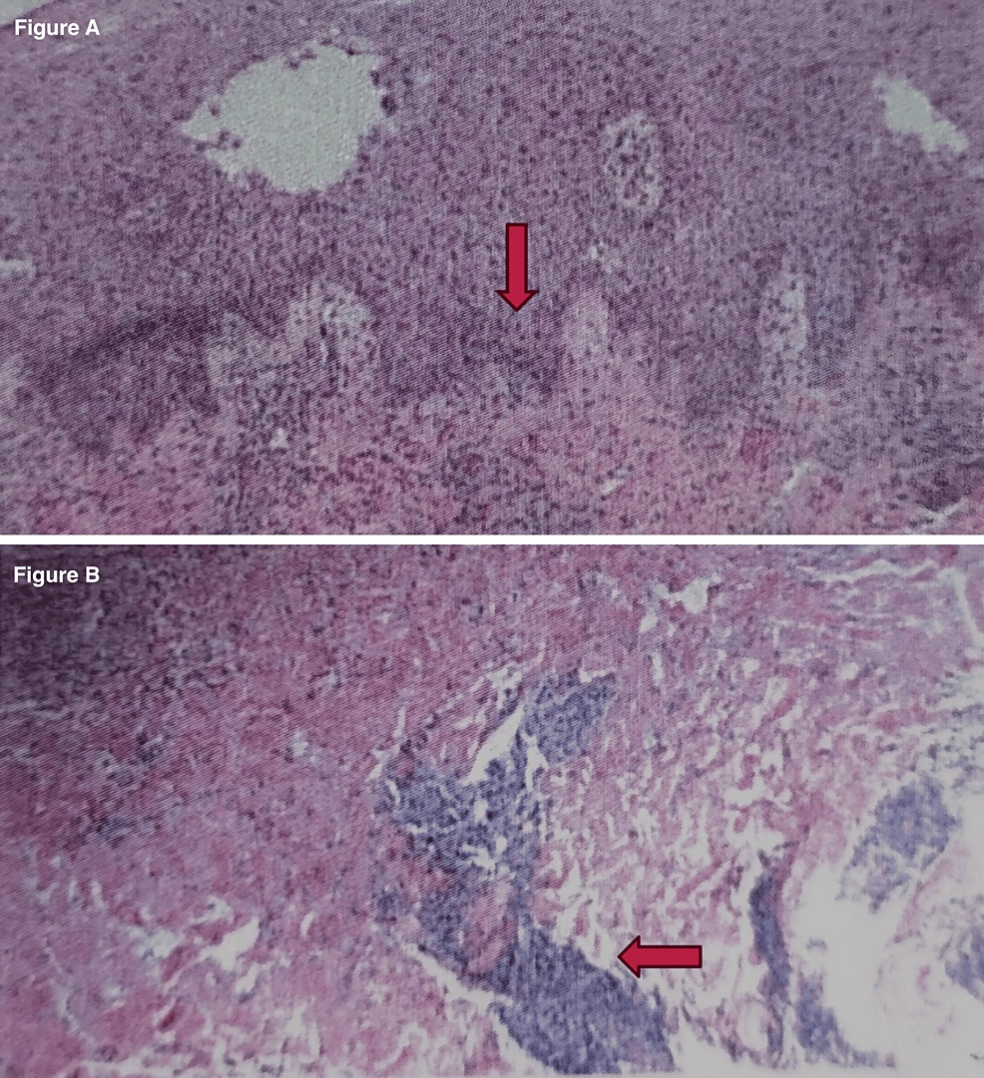
Figure 1: Skin biopsy (A) Neutrophilic dermatosis consistent with PG. (B) Neutrophilic infiltrate. PAS, GMS, and gram stains were done and were negative for infectious organisms. PAS, periodic acid-Schiff staining; GMS, Grocott methenamine silver; PG, pyoderma gangrenosum

The chest x-ray was unremarkable. A brain MRI with and without gadolinium was done to evaluate for severe neuro Behcet’s yielding normal results. A skin biopsy of the right leg ulcer showed acanthosis of the epidermis with associated spongiosis and neutrophilic infiltration in the stratum corneum and dermis consistent with a diagnosis of PG (Figure 1).

She was diagnosed with an exacerbation of BD and PG following COVID-19. Due to the severe headaches, arthralgias, and rapidly progressive skin ulceration on her right buttock and right lower extremity, she received a pulse of methylprednisolone 1000 mg intravenously (IV) daily for three days, followed by a daily dose of 60 mg of oral prednisone. Her azathioprine dosage was increased from 50 mg daily to 100 mg twice a day. She continued to receive infliximab at 560 mg IV every eight weeks. Local ulcer care was provided on a daily basis.

The malaise, fever sensation, arthralgias, and chills resolved within three days of starting treatment. Skin ulcers began to heal within five days. She was discharged home on the eighth hospital day on 60 mg oral prednisone daily, azathioprine 100 mg PO bid, infliximab 560 mg every eight weeks, and low-dose aspirin. Within four weeks of hospital discharge, headaches, arthralgias, and PG resolved, and prednisone weaning was started. The prednisone was stopped by the sixth month after discharge. At twelve months post-hospitalization, she continued off prednisone. Headaches, arthralgias, and skin ulcers had resolved. She returned to her job as an ED nurse one week after hospital discharge. 

## Discussion

We report a woman with PG as part of BD manifestations who after years in remission of both autoimmune diseases suffered a relapse of the BD and PG immediately following COVID-19 infection. The relapse was of such severity that she required admission to the hospital and treatment with high-dose steroids. The relapse of both BD and PG occurred even when she was being treated with robust immunosuppressive therapy consisting of IV infliximab and oral azathioprine, and after a COVID-19 infection with moderate symptoms, no pulmonary involvement and a short convalescence period.

Although there are studies investigating the course of COVID-19 in various rheumatologic disorders, knowledge about COVID-19 outcomes in BD is limited. In a retrospective cohort study of outcomes and rate of Behcet’s exacerbation in patients after COVID-19 infection, the most common BD symptoms following COVID-19 were fever and myalgia. Of the study participants, 47% had headaches, 42% had dyspnea, and 47% had arthralgia [21]. In the study of Espinosa et al., headache was present as a COVID-19-induced BD exacerbation symptom in three (75%) patients, while dyspnea and arthralgia were not observed [22]. Indeed, distinguishing between new symptoms related to COVID-19 and BD exacerbation can be challenging, as both conditions can present with overlapping symptoms such as fever, fatigue, and respiratory symptoms. Additionally, considering the patient’s medical history, including any previous patterns of BD and PG exacerbations and COVID-19 symptoms, can help guide the diagnostic process. 

Case reports describing PG exacerbations and PG-like skin lesions in subjects who were infected with COVID-19 have been published [23]. Similar to our patient, these case reports describe the development of painful ulcers a short time after COVID-19 infection. Pathological findings of lesion biopsies in the case reports were consistent with a neutrophilic dermatosis as seen in PG. Treatment with high-dose steroids and anti-tumor necrosis factor inhibitors was started due to the severity of the PG, mirroring the approach used in our patient. 

PG associated with BD has been reported [3-15]. It has been suggested that both diseases share common pathogenic pathways and proinflammatory cytokine profiles. Overactive innate immune responses in PG with an increase in serum levels of granulocyte colony stimulation factor, IL-1, IL-6, IL-8, CXCL 1,2,3,16, and RANTES are seen [24,25]. In BD the adaptive and innate immune systems are active with an increase in serum levels of IL-1, IL-6, IL-17, tumor necrosis factor-alpha (TNF-α), interferon-gamma, and granulocyte-macrophage colony stimulator factor. In addition, both PG and BD are considered to be autoinflammatory syndromes characterized by episodes of disease activity when the cytokine environment is proinflammatory [26].

COVID-19 infection and other pathogenic coronavirus such as SARS-Cov-1 and MERS favor a strong proinflammatory milieu when there is a maladaptive innate immune system response [27]. Several mechanisms of maladaptive innate immune responses have been reported. Autoantibodies directed against type I interferon block its immune effects, resulting in a decreased antiviral response. Deregulation of the C3 cascade increases inflammation through mechanisms such as the release of neutrophil extracellular traps. The lack of activation of Toll-like receptor 7 in plasmacytoid dendritic cells by COVID-19 results in a decreased interferon release, leading to an increase in COVID-19 viral load [27]. Also, there are increased levels of TNF-α, IL-6, IL-1 and proinflammatory cytokines, which have been reported in COVID-19-infected subjects [28-29]. Thus, COVID-19 infection in the context of a maladaptive innate immune response may exacerbate PG through a proinflammatory environment that stimulates an autoinflammatory disease.

There have been several studies reporting skin adverse events after COVID-19 vaccination, including PG [30-34]. This is not the case in our patient since her last COVID-19 vaccination was one year prior to her disease relapse. A case has been documented in the literature where PG was diagnosed subsequent to COVID-19 infection [35].

## Conclusions

In conclusion, this case highlights the need to consider that development or relapse of an inflammatory, autoimmune disease may occur following COVID-19. This may occur even in infected patients with mild to moderate symptoms, of short duration and rapid convalescence. An early diagnosis and institution of appropriate therapy will decrease morbidity. In our subject with BD and PG associated with BD, both diseases relapsed. High-dose steroids and adjustments in the immunosuppression helped achieve disease remission and rapid tapering of the corticosteroid therapy.

To our knowledge, this is one of the rare documented cases in the medical literature of the coexistence of BD and PG, where both diseases exacerbate after COVID-19 infection. Further research is needed to better understand the relationship between COVID-19 infection and autoimmune diseases in order to develop strategies for the prevention and optimal management of this complication. 
